# Simulating preventative testing of SARS-CoV-2 in schools: policy implications

**DOI:** 10.1186/s12889-020-10153-1

**Published:** 2021-01-12

**Authors:** Ali Asgary, Monica Gabriela Cojocaru, Mahdi M. Najafabadi, Jianhong Wu

**Affiliations:** 1 Disaster & Emergency Management, School of Administrative Studies and Advanced Disaster, Emergency and Rapid-response Simulation, York University, Toronto, Canada; 2 Department of Mathematics & Statistics, University of Guelph, Guelph, Ontario Canada; 3 Advanced Disaster, Emergency and Rapid-response Simulation, York University, Toronto, Canada; 4 Canada Research Chair in Industrial and Applied Mathematics, Laboratory for Industrial and Applied Mathematics (LIAM), York University, Toronto, Ontario Canada

**Keywords:** COVID-19, Agent-based Modelling, COVID-19 testing, School testing, Disease modelling

## Abstract

**Background:**

School testing for SARS-CoV-2 infection has become an important policy and planning issue as schools were reopened after the summer season and as the COVID-19 pandemic continues. Decisions to test or not to test and, if testing, how many tests, how often and for how long, are complex decisions that need to be taken under uncertainty and conflicting pressures from various stakeholders.

**Method:**

We have developed an agent-based model and simulation tool that can be used to analyze the outcomes and effectiveness of different testing strategies and scenarios in schools with various number of classrooms and class sizes. We have applied a modified version of a standard SEIR disease transmission model that includes symptomatic and asymptomatic infectious populations, and that incorporates feasible public health measures. We also incorporated a pre-symptomatic phase for symptomatic cases. Every day, a random number of students in each class are tested. If they tested positive, they are placed in self-isolation at home when the test results are provided. Last but not least, we have included options to allow for full testing or complete self-isolation of a classroom with a positive case.

**Results:**

We present sample simulation results for parameter values based on schools and disease related information, in the Province of Ontario, Canada. The findings show that testing can be an effective method in controlling the SARS-CoV-2 infection in schools if taken frequently, with expedited test results and self-isolation of infected students at home.

**Conclusions:**

Our findings show that while testing cannot eliminate the risk and has its own challenges, it can significantly control outbreaks when combined with other measures, such as masks and other protective measures.

## Background

Schools bring together a large number of students with very wide social connections and networks in a closed environment where they share spaces, facilities, and equipment [1]. It is argued and expected that reopening and operations of schools may reinforce the virus spread and thus increase the number of cases both in the schools and in the community. Without testing and gradual relaxation of social distancing, a second wave could occur during the school year [2]. However, given the importance of learning that takes place inside the schools and the inability of working parents to supervise remote learning at home, there are also concerns regarding school closures and full switch to distant learning. To minimize the impact of the pandemic and to create a safe environment for students while continuing school operations, public health agencies have provided schools with reopening guidelines and procedures that mainly focus on social distancing, hygiene practices, screening and monitoring, and reporting in close collaboration with families [3–7].

Testing has been an important element of the COVID-19 pandemic management [8]. Thus, while many schools are currently implementing on-site symptom screening [5], testing may become an additional preventative tool depending on how the pandemic progresses. Testing students prior to and during the school session has become a public health policy and planning issue, as well as a challenge. According to the European Centre for Disease Prevention and Control (ECDC), “*a well-implemented testing strategy in school settings might play an important role in preventing virus transmission within the school setting and to the community*” (p.2) [1]. However, because there is no consensus on school testing, because testing is costly and is perceived as painful or uncomfortable, and because it requires significant preparation and planning, it is vital to analyze and assess the outcome and effectiveness of different testing scenarios in controlling disease outbreaks. Simulation modeling can help with exploring and examining the impacts of different testing scenarios. Although testing capacities are currently limited, as technology enhances, it is argued that systematic testing may help in controlling the outbreaks by identifying pre-symptomatic and asymptomatic individuals, while allowing schools to continue their activities. This is also in line with arguments that challenge the current testing strategies which focus on testing symptomatic individuals [8]. This paper describes and presents sample results of an agent-based simulation tool that is developed to help decisionmakers examine different school testing strategies and scenarios. The simulation results are validated with the help of a theoretical viewpoint on the effective reproduction number of the virus in a school environment, which shows that contact tracing, mask wearing and social distancing within classrooms are helping in decreasing the testing frequency and overall test numbers needed to control outbreaks.

### School testing challenges

To test students and staff in elementary and secondary schools, some challenges and issues should be addressed: 1) the need for conducting significant number of tests for finding a small number of infected positive cases; 2) testing, especially frequent swab testing, may cause some pain or discomfort for children-although progress has been made towards less painful tests, such tests are not widely available; 3) almost all currently available tests have significant number of false-negative and false-positive rates [9, 10] that can cause confusion and stress; 4) tests may create a false sense of security among those tested; 5) conducting regular testing in schools is logistically challenging and requires additional resources, planning and operational burdens; 6) large scale testing of students may add more pressures to local public health testing capacities, and in particular, adding school testing may create more issues and increase waiting time for tests and test results; 7) tests are costly and the current PCR tests require specialized machinery and supply- more tests translates into more costs that need to be justified against the benefits that they generate; 8) the time between collecting a sample and receiving the test results is often too long-currently the test results are available after 1 to 6 days depending on the type of test and how many tests are being conducted [9], because the main goal of testing is to identify potential carriers and isolate them and those in close contact with them. As the time between sample collection and test results increases, the effectiveness of tests diminishes (For example, in the Province of Ontario, Canada, test results are returned between 1 to 3 days under normal conditions but this interval can increase to up to a week or even more under busy conditions); 9) public acceptance of mass testing particularly for younger kids in schools is low which may create push back; for example, a study conducted by Statistics Canada found that only 4 in 10 people support mandatory random COVID-19 testing and older adults are more supportive of this idea compared to younger people [11]; 10) privacy issues may arise in school testing-although the tests results can be kept private, subsequent follow up actions such as the temporary exemption from school, may disclose the identity of infected students. A summary of the challenges above is depicted in Table 1.

**Table 1:** Summary of School Testing Challenges

1	Significant number of tests should be performed to find a small number of infected positive cases
2	Tests may cause pain or discomfort, especially for children
3	Available tests have significant false-negative and false-positive rates that can cause confusion and stress
4	Tests may create a false sense of security among those tested
5	Regular testing in schools would be logistically challenging and requires additional resources, planning and operational burdens
6	Large-scale testing of students may add more pressures to local public health testing capacities and may increase overall testing waiting times
7	Tests are costly; more tests translate into more costs that need justification over the benefits they generate
8	The delay before test results are available can reduce testing effectiveness because the main goal of testing is to identify and isolate potential viral vectors and those in close contact with them
9	Public acceptance of mass testing particularly for younger kids in schools is low which may create push back
10	Privacy issues may arise in school testing, as subsequent follow up actions may disclose the identity of infected students

### School testing benefits

According to the ECDC (2020) the objectives of school testing are: “*1) to ensure early identification of cases among students and staff in order to conduct contact tracing and initiate prevention and control measures, thereby reducing further transmission; 2) to identify infection in students and staff at high risk of developing severe disease due to underlying conditions; and 3) to support investigations and studies concerning the role of children in the transmission of COVID-19*.” (ECDC, 2020, p. 1). As such, school testing provides some benefits including: 1) more information about the disease status in the community and its subsystems- in the absence of clinical solutions such as a vaccine or a drug, testing enables early detection of infected students and prevents outbreaks, and thus is one of the most powerful tools for managing the pandemic as it allows to identify the infected individuals earlier to reduce additional infections; 2) although testing is uncomfortable and may be painful for some people, it is less painful than hospitalization; 3) testing can reduce pressures on different stakeholders, particularly mental pressures on parents and teachers by providing them with some reassurance that schools are being monitored [12]; 4) asymptomatic cases, that are mainly among the younger ages and able to spread the virus, can only be detected through random testing; 5) testing enables schools to continue their operations with lower risks and uncertainty: without testing, schools may have to go to lock down more frequently, most likely because when too many cases are identified by screening, it will be too late to control the outbreak without closing the school. Moreover, if the pandemic continues, closure of schools may not be a long-term solution; 6) without regular testing, co-infection or overlap between influenza and COVID-19 can create more chaos, particularly during the flu season when it will be difficult for parents or those who screen the children for symptoms, to identify most likely COVID-19 symptoms. A summary of the challenges above is depicted in Table 2.

**Table 2:** Summary of School Testing Benefits

1	Allows to identify the infected individuals earlier to reduce additional infections
2	It is less painful than hospitalization for those who would have been infected in the absence of testing
3	It can reduce pressures on different stakeholders, particularly mental pressures on parents and teachers
4	Asymptomatic cases, that are mainly among the younger ages and able to spread the virus, can only be detected through random testing
5	It enables schools to continue their operations with lower risks and uncertainty, which is important because as the pandemic continues, closure of schools may not be a long-term solution
6	without regular testing, co-infection or overlap between influenza and COVID-19 can create more chaos, particularly during the flu season when it will be difficult for parents or those who screen the children for symptoms, to identify most likely COVID-19 symptoms

### School testing strategies

Public health agencies are undertaking or considering many different strategies with regard to school testing. These include: 1) Random regular testing in which all or a sample of students and schools are tested regularly: For example, the Province of Saskatchewan, Canada has announced that students with parental consent will have the option to participate in random testing [12]. These tests can be integrated into routine in-school childhood vaccinations. Selection of schools to be tested could be based on the surge in communities where schools are located. 2) One-time testing of all students and teachers returning to schools after summer holidays or school closure: This strategy aims to ensure that students come to school uninfected. For example, the Province of Alberta, Canada, aimed to test all students and teachers before reopening the schools [12]. This would need prioritization of testing for students and teachers before school reopening. The Province of Saskatchewan also encourages teachers and education staff to get tested prior to the school year; 3) Testing students and teachers with symptoms: this is in line with the current practice in which suspected students and teachers are requested to be tested. Most of the current guidelines provided by Public Health agencies to schools advise them to recommend students or teachers with COVID-19 symptoms to self-quarantine and get tested. According to this strategy, testing does not have to be done in schools, but provisions can be made to give priority to such tests at designated testing sites. Table 3 summarized these different school testing strategies.

**Table 3:** Summary of Pros and Cons of Different School Testing Strategies

Testing Strategy	Major Pros	Major Cons
Random regular testing in which all or a sample of students and schools are tested regularly	Can be integrated into routine in-school childhood vaccinations	Large samples maybe needed to be effective
One-time testing of all students and teachers returning to schools after summer holidays or school closure	Can help reducing the anxiety of reopening among the students, schools’ staff and parents	Lsrge number of tests to be processed at a given time and may not prevent contamination after testing.
Testing students and teachers with symptoms	Small number of tests are required.	Detects symptomatic cases only

In this paper we focus on the first strategy that is based on the regular random testing in all or some of the schools. This strategy could have several options and scenarios depending on the frequency of testing (daily, weekly), number of days it takes to have test results, test expiry days, and actions taken subsequent to positive test results such as intensified testing or self-isolation of infected classes.

## Method

**Fig. 1:**
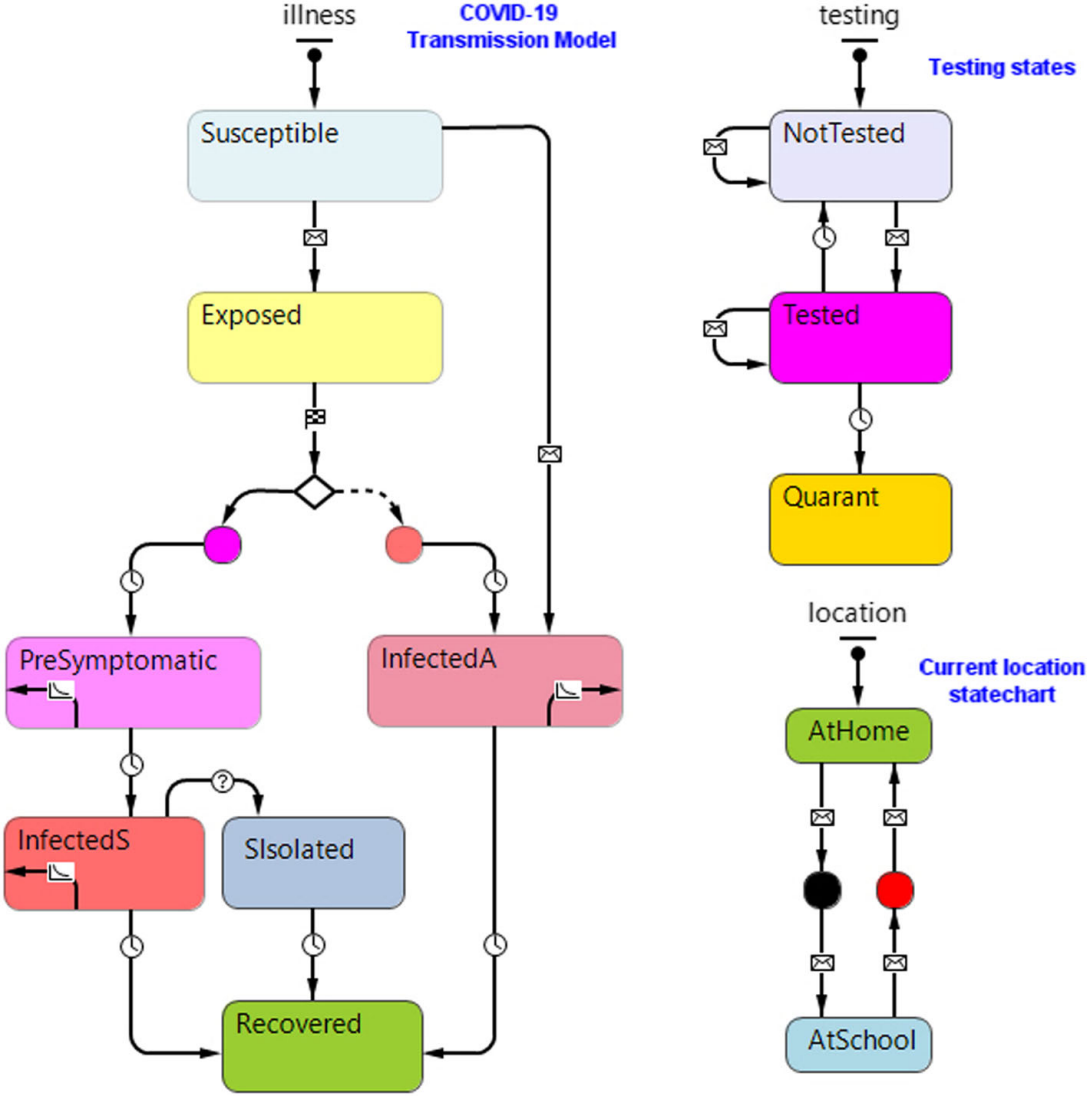
Student agent state charts (disease transmission-left, testing-top right and location-bottom right)

We developed a modeling and simulation tool to assess the outcomes of regular random school testing. We use an agent-based model with two agents of *Student* and *Class*. This model does not include *Teacher* and *Staff*, although the teacher population can be included in the class population adjusting for appropriate contact rates and transmission levels. A modified SEIR model captures the disease transmission (Fig. 1). Students go to their school in the morning of weekdays and return home after the end of the school hours. This movement is captured by the *location* state chart. If a student tested positive, he or she stays home. As shown in the *illness* state chart, each student is at Susceptible state before it is exposed to a random asymptomatic infected classmate. After the exposure, the student may become infectious with different probabilities, either pre-symptomatic or asymptomatic. Transmission rate is defined as a product of *transmission probability* and *number of contacts* when the student resides at school. The model allows for a portion of infected symptomatic cases to be self-isolated. Both asymptomatically and symptomatically infected students recover within a specified number of days. In this model, we have assumed that recovery from the infection would confer lifelong immunity (e.g., within the life span of the modeling), and thus, once an agent is recovered from the infection, it cannot get infected with this virus another time.

The *testing* state chart captures the testing states of student agent. Students are all in NotTested state before being randomly tested. As students are tested their state changes to Tested. They will remain in this state and the school until the test result is provided. If the test result is positive, the student is self-isolated at home (Quarant state), and if not, the student will go back to NotTested state after the test expiry date and the student can be retested. While in Self Isolated state due to symptoms checking (SIsolated) or due to testing (Quarant) states, students stay at home.

The *Class* agent defines the location of students in the school and infectious status of the class. Each class contains its own students throughout the simulation. In other words, students of each class are always together. Once a student in a class becomes infected, it starts infecting others. As testing starts, a subgroup of the class is created to account for tested students.

The transition from Susceptible to InfectedA state is solely for model initialization that allow us to enter one or more infected students at the beginning or at an arbitrary stage of the modeling process. The initial number of infected students can be decides based on the prevalence rate in the community in which the school is operating.

**Fig. 2:**
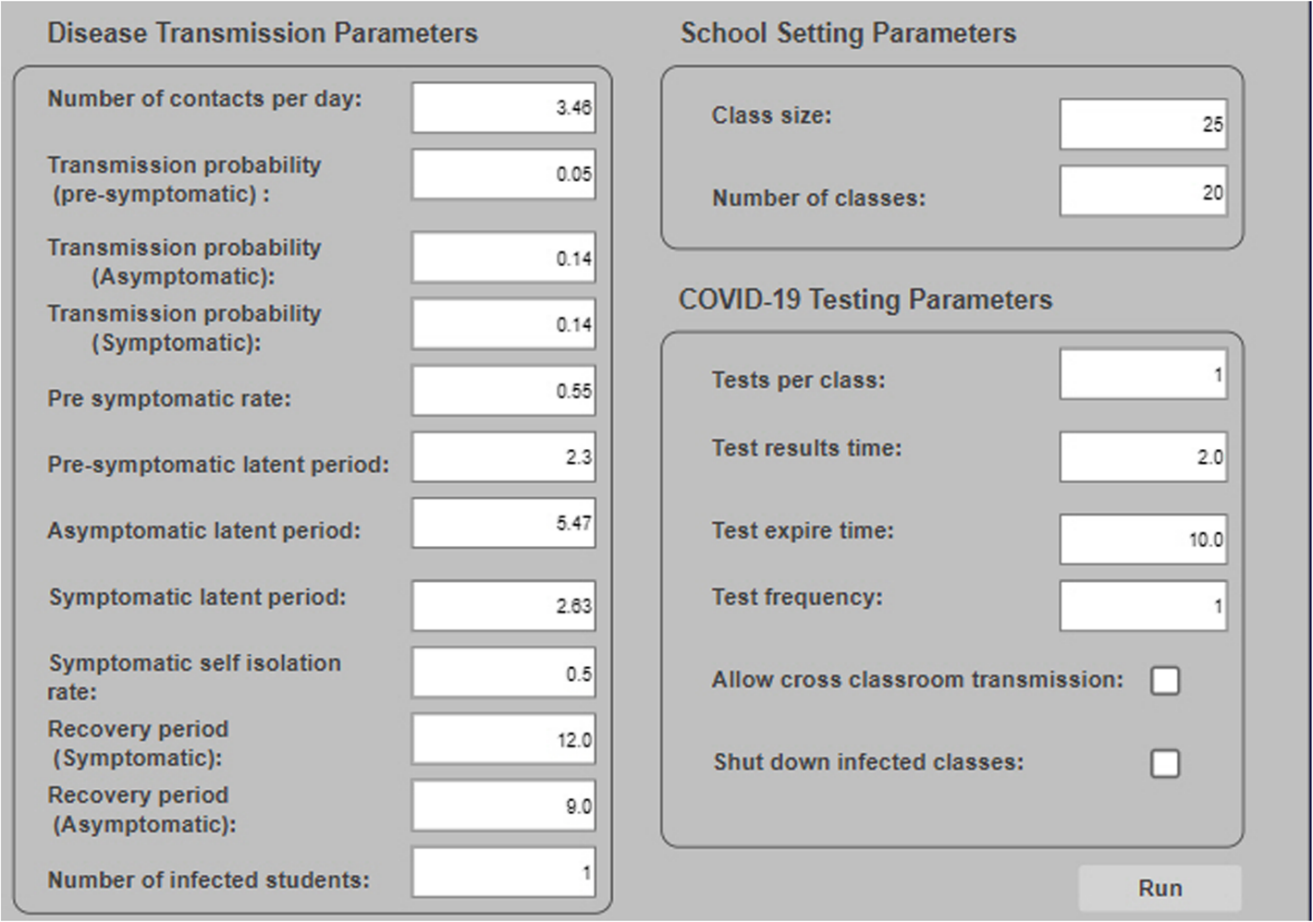
Parameter setting page of the school testing simulation tool

**Fig. 3:**
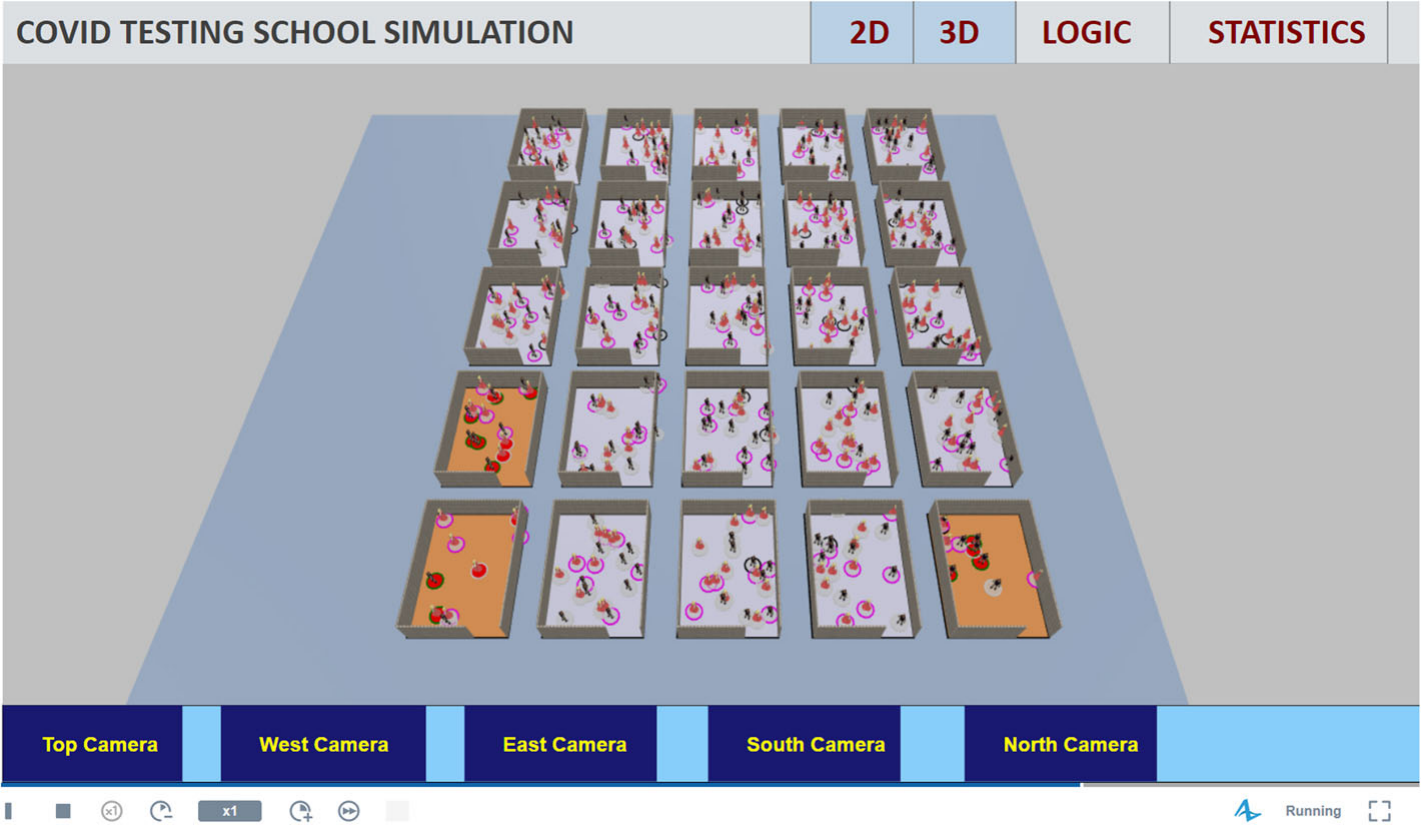
3D animation page of the simulation

The simulation tool includes a parameter setup page (Fig. 2), a 3D animation window (Fig. 3), a 2D visualization, and a statistics section that presents the simulation results.

## Results

The simulation tool allows users to set relevant values for each parameter, and thus examine different scenarios. We run the model for a baseline parameter setting (Table 4) to demonstrate the simulation results. While parameters can be localized as needed, here we set the parameters based on Ontario information as reported in the existing literature.

**Table 4:** Parameters setting for the base model

Parameter Type	Parameters	Value
**Disease related**	Number of contacts per school day [13]	3.45
	Transmission probability of pre-symptomatic [14]	0.05
	Transmission probability of symptomatic [15]	0.14
	Transmission probability of asymptomatic [15]	0.14
	Pre-symptomatic rate (portion) [15]	0.45
	Pre-symptomatic latent period day) [14]	2.3
	Asymptomatic latent period (day) [14]	5.47
	Symptomatic latent period (day) [14]	2.63
	Self-isolation rate [14]	0.5
	Symptomatic recovery period (day) [16]	12.0
	Asymptomatic recovery period (day) [16]	9.0
	Number of initially infected students	3
**Class related**	Class size	25
	Number of classes	20
**Test related**	Number of tests in each class	1
	Test results time (day)	2
	Test expiry time (day)	10
	Test frequency (day)	10

We run the base model for a school with 20 classes and 25 students in each class for a total of 500 students. We also assume that the simulation starts with three asymptomatic infected students. Simulation results are presented using Monte Carlo simulations with 500 iterations to capture the stochastic variations and randomness nature of testing. The simulation period is 60 days.

### Infections and tests - daily tests

**Fig. 4:**
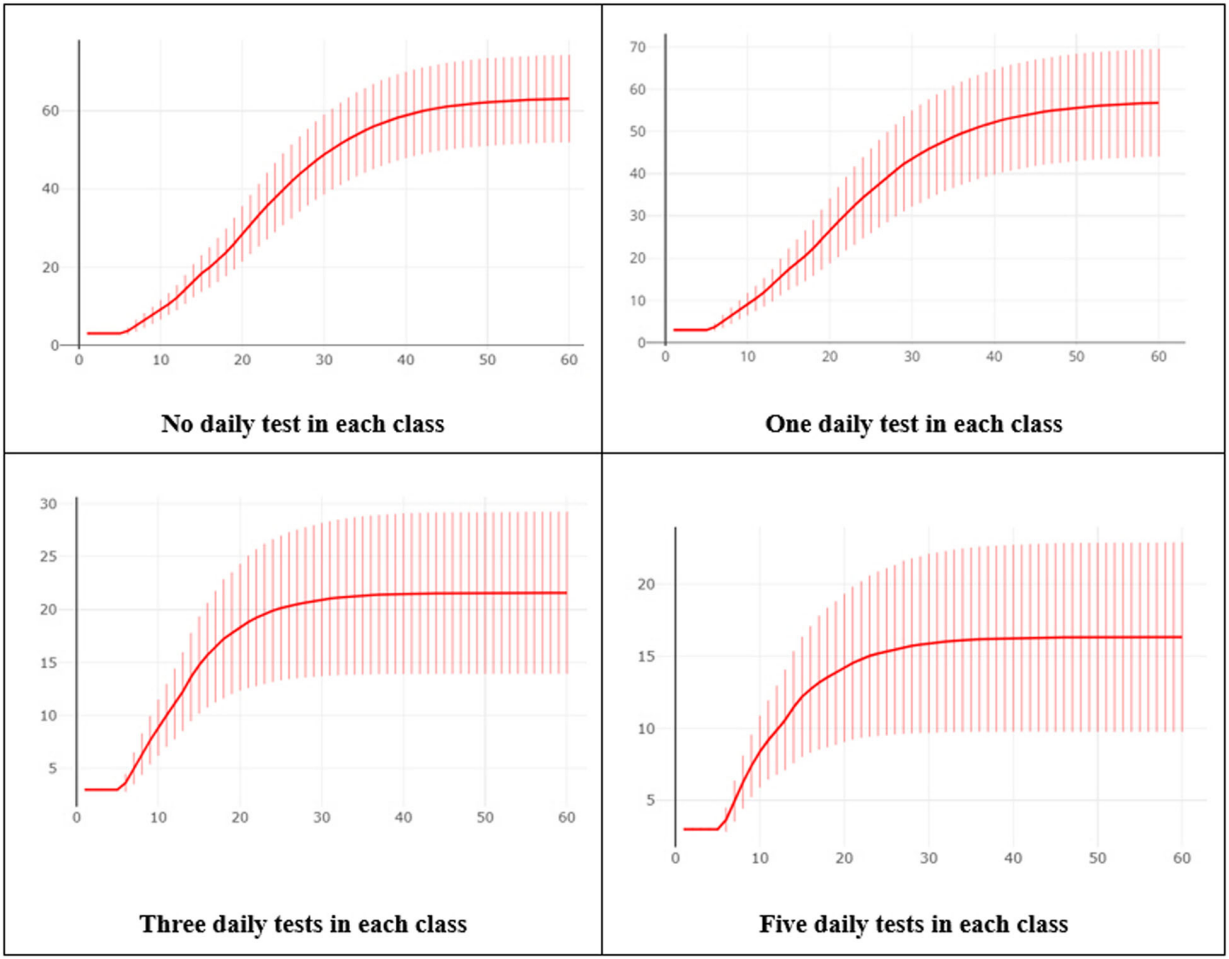
Number of infected students under different number of daily tests at base parameters setting (Monte Carlo Simulation of the model with 500 iterations)

Figure 4 shows the accumulated number of infected students (symptomatic and asymptomatic) under different number of daily tests in each class. As expected, these results show that increasing the number of tests reduces the number of additional infections. However, interestingly, the results show that increasing the number of daily tests beyond 5 tests per class (as high as 20% percent of the pupils in the class) under the current parameter setting, would not make a significant difference.

**Fig. 5:**
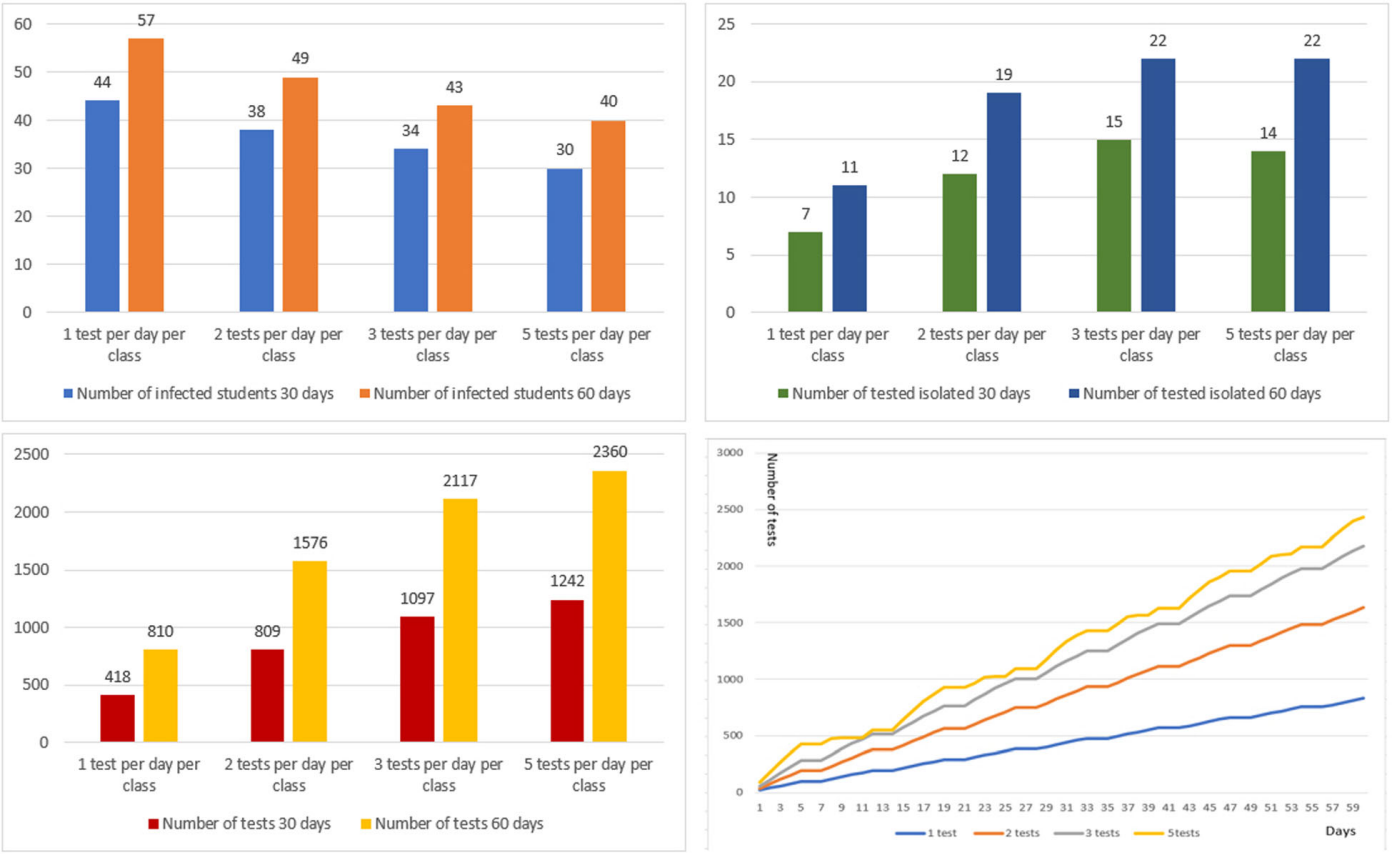
Number of infected students (top left), number of tested isolated students (top right), tests needed (bottom left), and accumulated number of tests (bottom right)

Figure 5 Shows the number of tests, number of infected students, and number of students isolated through testing at day 30 and day 60.

### Infections and tests - weekly tests

**Fig. 6:**
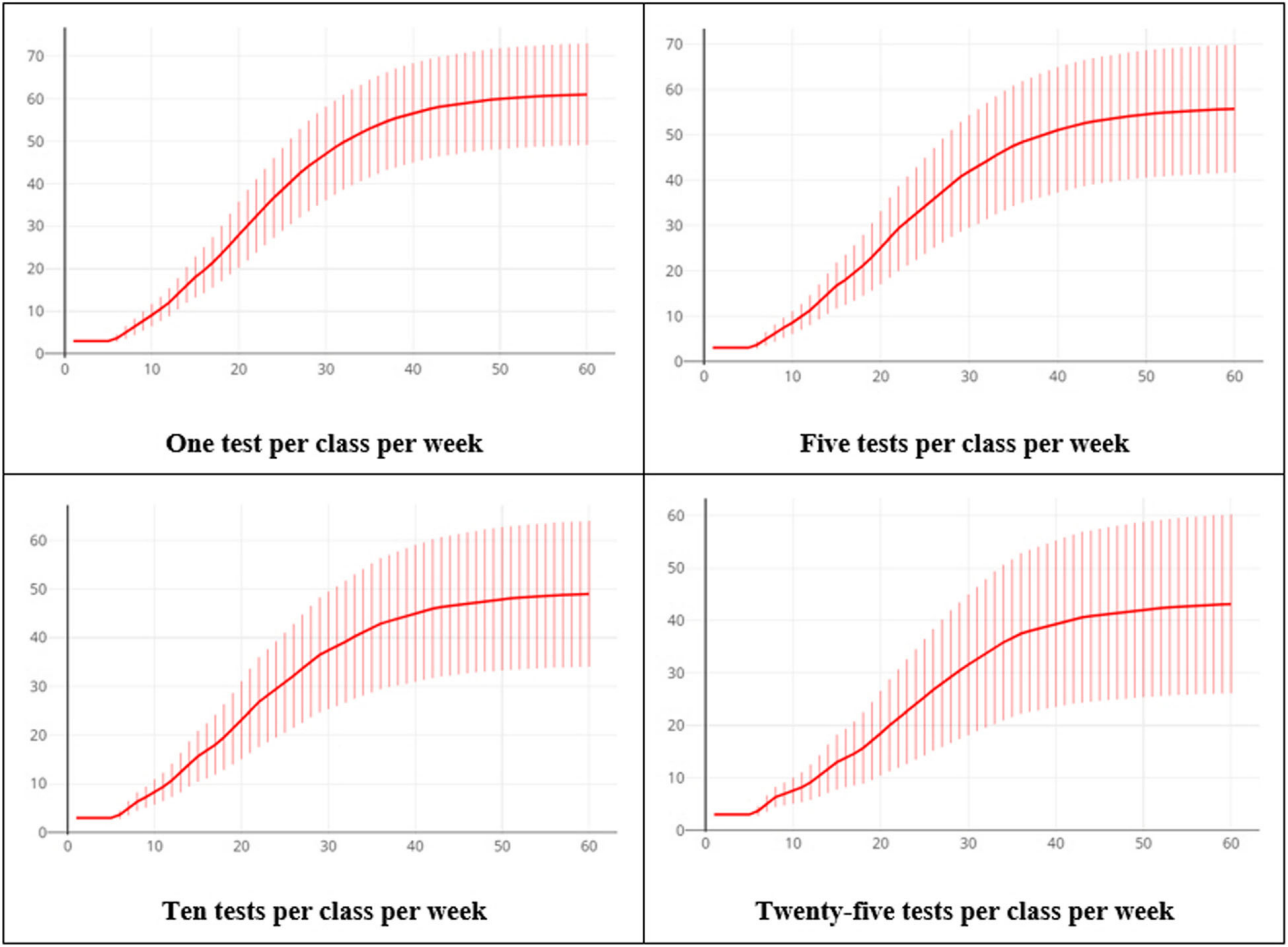
Accumulated number of infected students under different number of tests per week

Figure 6 presents the simulation results for the base model considering a weekly test schedule. In this experiment, we compare the accumulated number of infected students based on number of tests per class per week. Although increasing the number of tests in each week can reduce number of cases, it does not seem to be as effective as daily tests for less than the whole class per week.

**Fig. 7:**
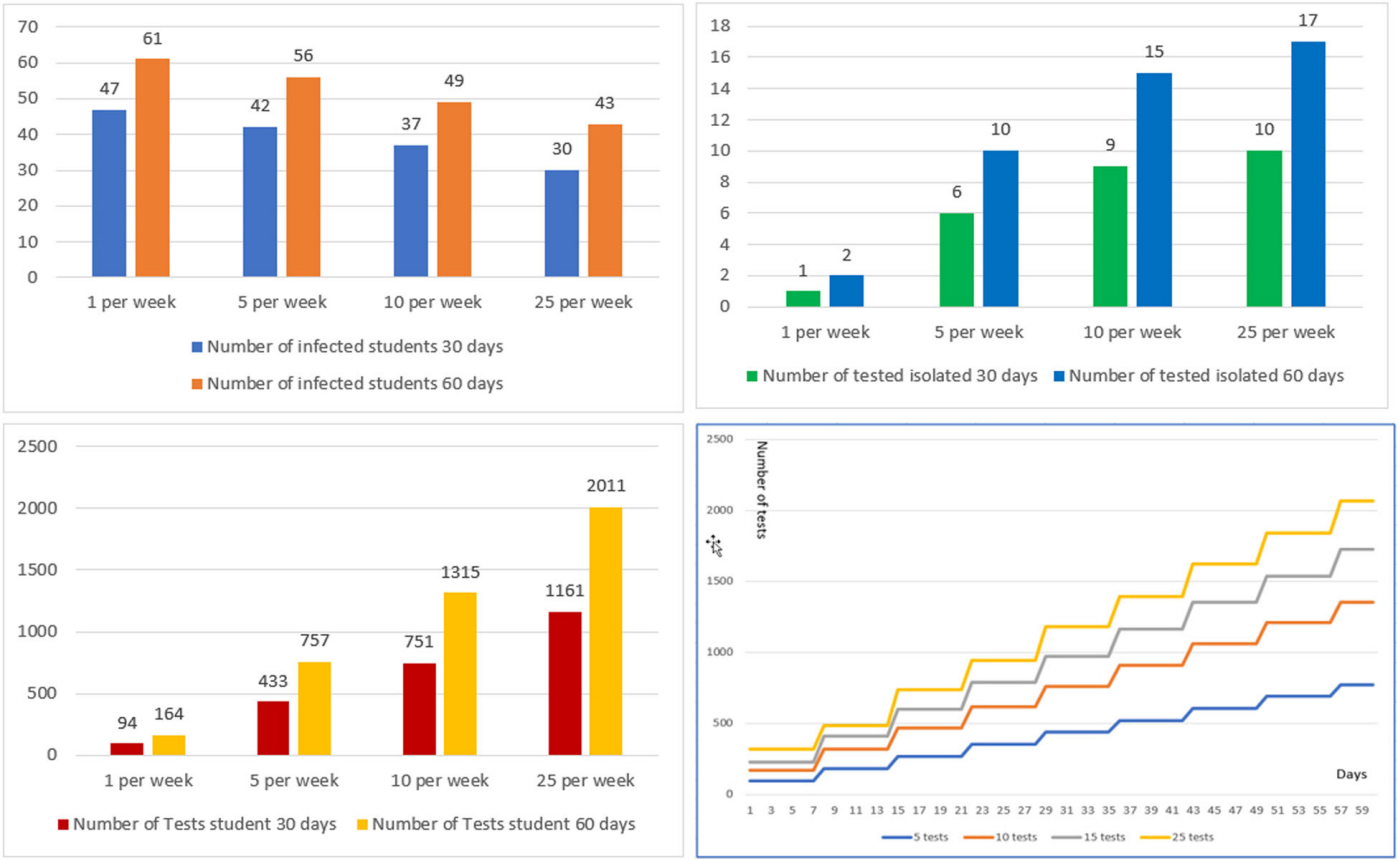
Number of infected students (top left), number of tested isolated students (top right), tests needed (bottom left), and accumulated number of tests (bottom right)

Figure 7 shows number of tests, number of infected students, and number of students isolated through testing for weekly testing strategy at day 30 and day 60 of the simulation.

### Infections and tests - test results days and test expiry days

**Fig. 8:**
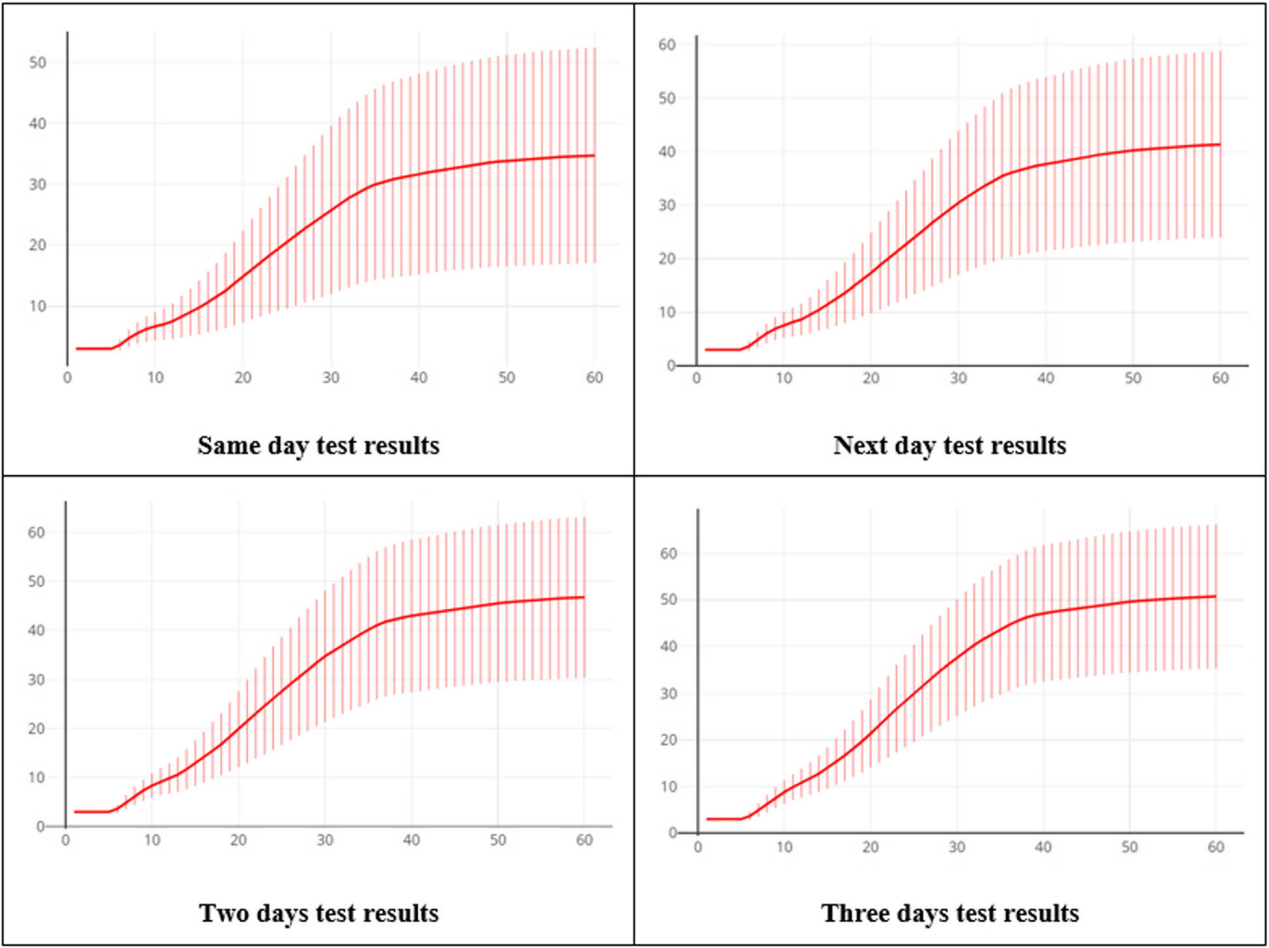
Simulation results for the base model parameters under different test results waiting times

Figure 8 shows the simulation results for the base model under different waiting days for test results. We compare same day test results with test results provided after 1, 2 and 3 days. As expected, providing the test results on the same day yields a lower number of cases as it expedites the isolation of confirmed positive cases, before they have time to spread the virus further in the school.

**Fig. 9:**
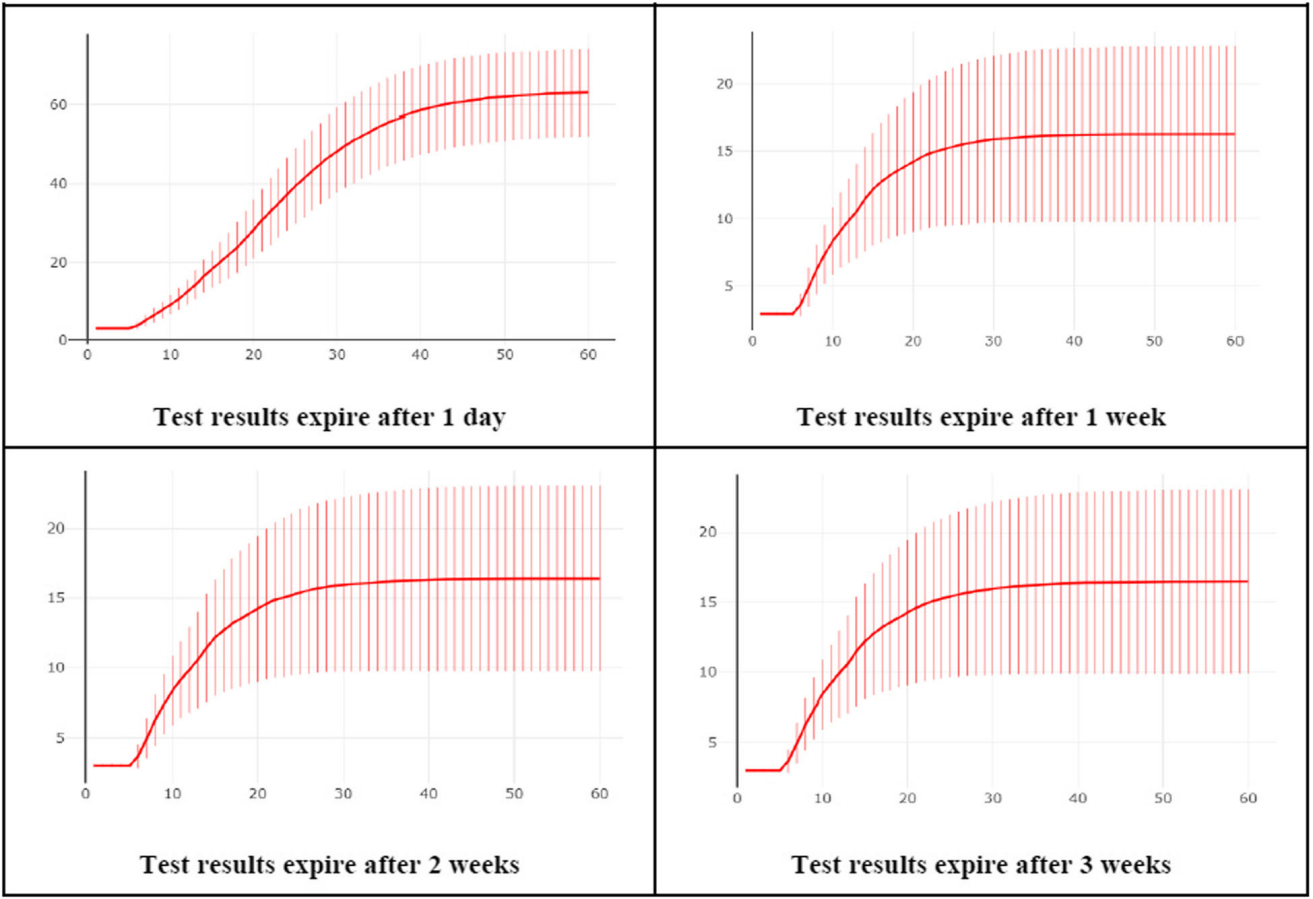
Simulation results for base parameters under varying test expiry days

Test results can be valid for different days before a student needs to be tested again. Test expiry period refers to the days by which a tested student would not be included in the testing. We ran the simulation to understand what the impacts would be setting different test expiry days. Figure 9 present the simulation results for the base model with varying days for test expiry. As expected, earlier expiry of the test reduces the number of detected infected cases, as tested students are added to the tested group, the probability of finding the actual infected students is reduced.

### Infections and tests – isolating students in infected classes

**Fig. 10:**
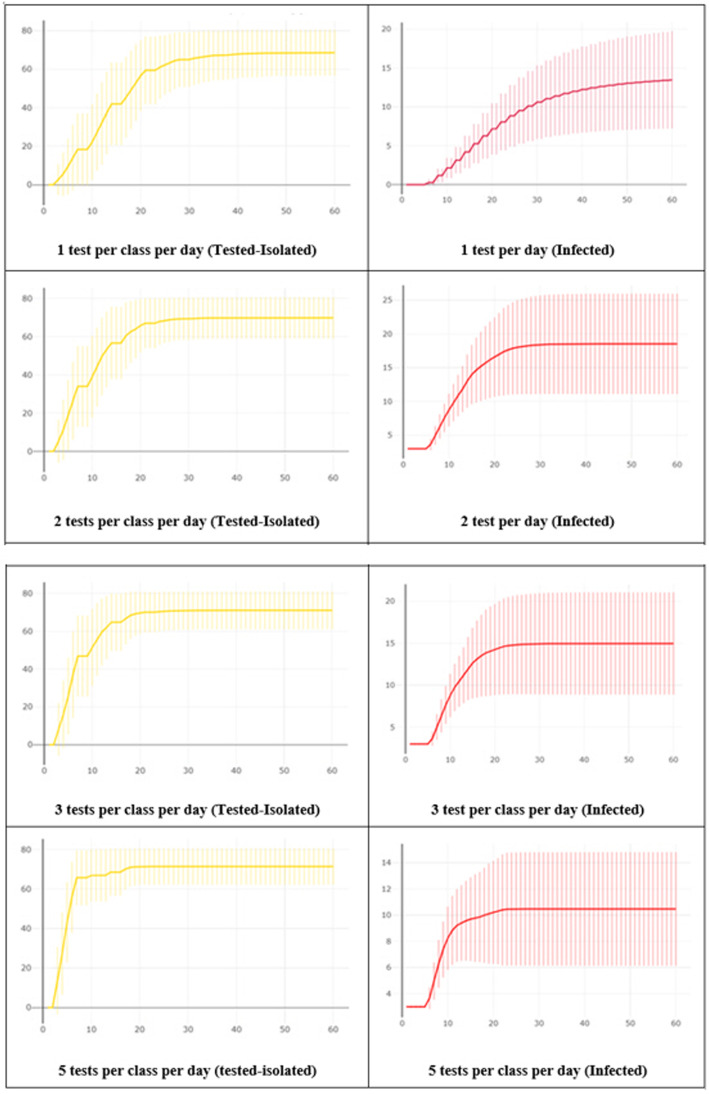
Number of students isolated (left) and infected (right) under different number of daily tests in each class when all students in infected class are self-isolated at home

**Fig. 11:**
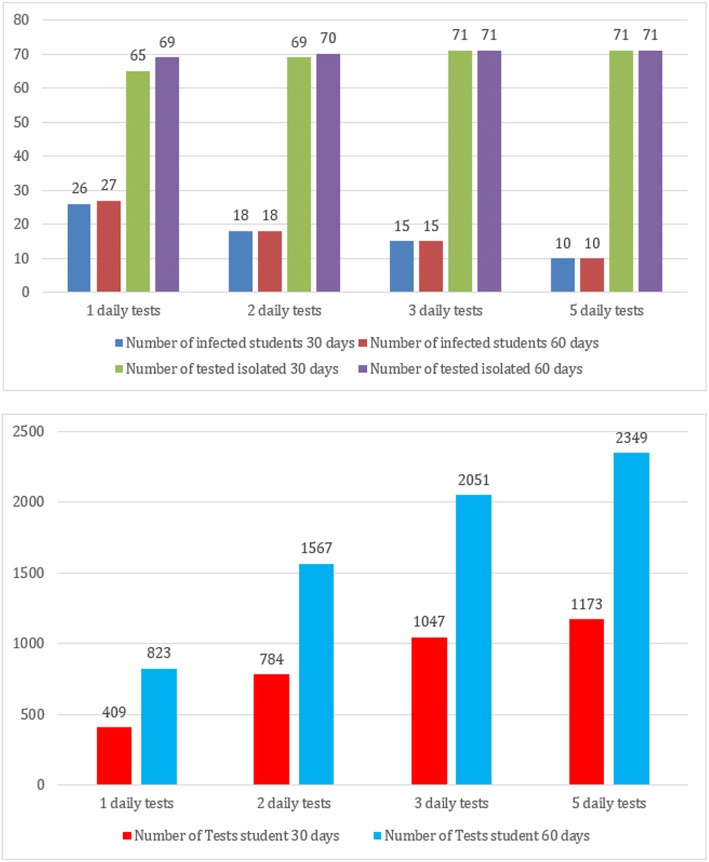
Total number of tests under different number of daily tests and when all students in infected classes are self-isolated at home

One important infection control strategy for the school would be to ask all students from classes with a positive test result to stay home for 14 days. This strategy assumes that in a close classroom the likelihood of infection among the classmates staying together for the school hours and days before the infected student is identified would be very high. Figure 10 and Fig. 11 present the number of students that will be isolated and the total number of infected students under this strategy. According to these results while the total number of isolated students will be similar under different number of tests per day, the total number of infected students decreases as more daily tests are conducted.

### Infections and tests -testing all students in infected classes

**Fig. 12:**
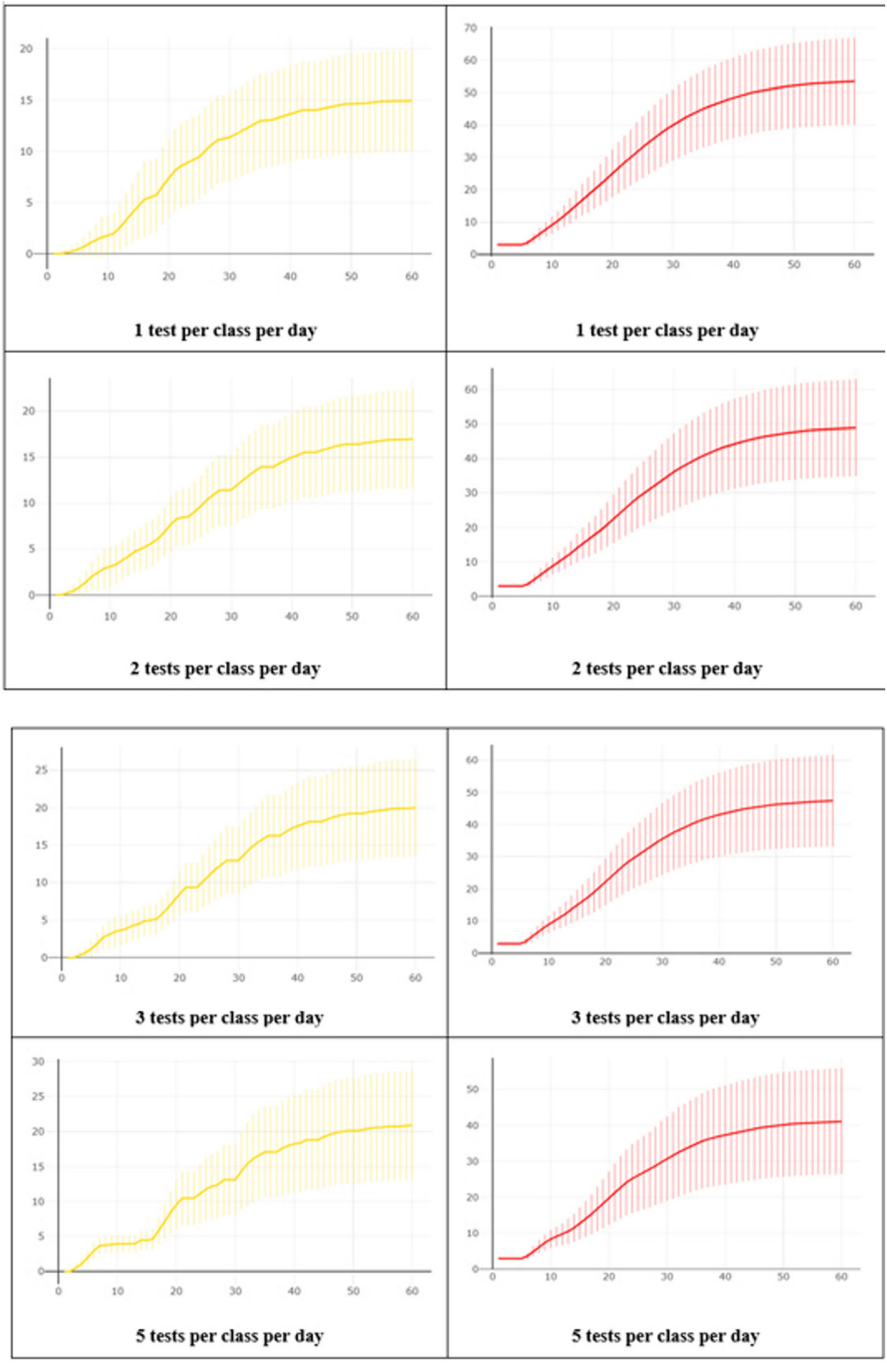
Number of students isolated (left) and infected (right) under different number of daily tests in each class when all students in infected classes are tested

**Fig. 13:**
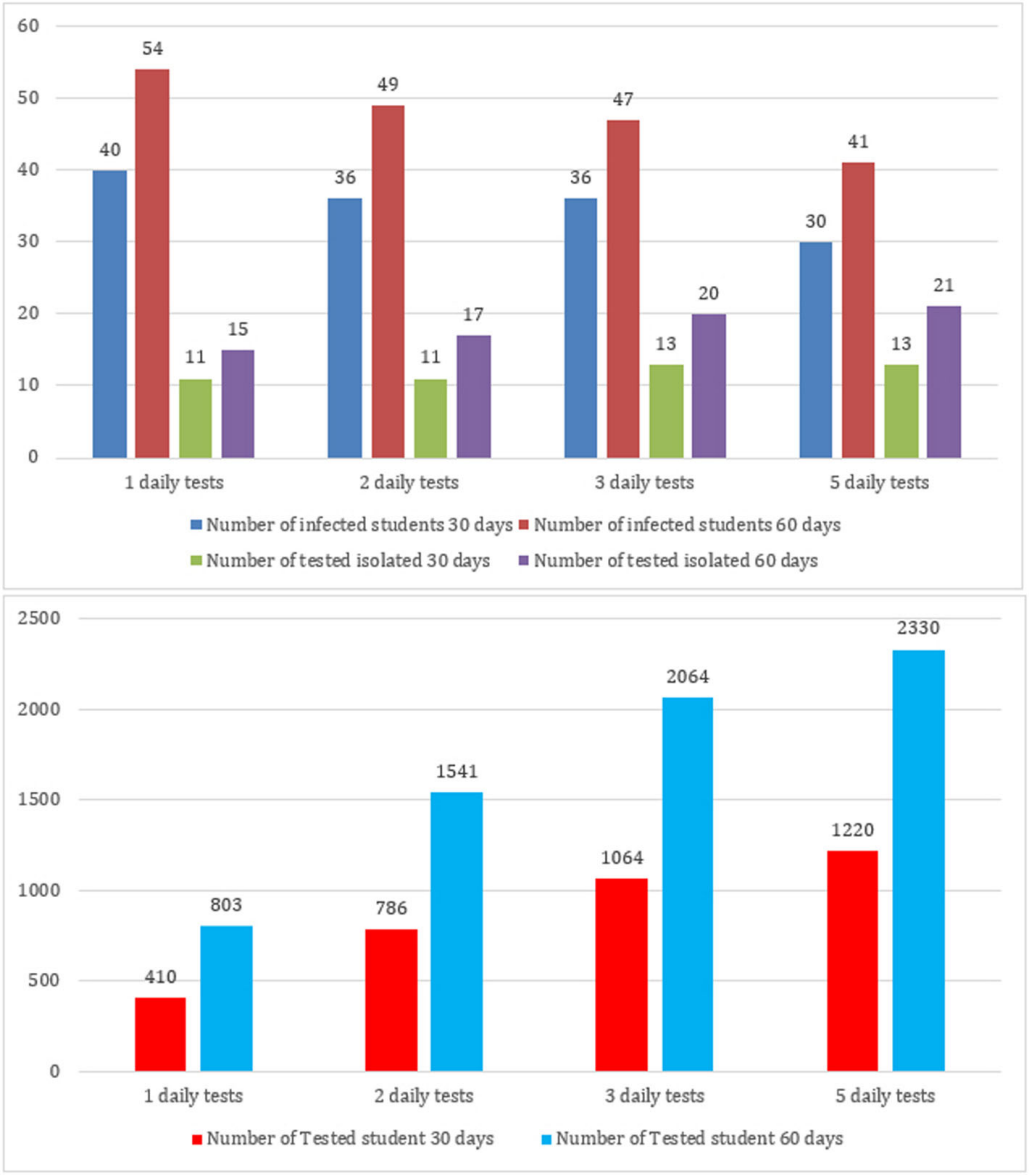
Total number of tests under different number of daily tests and when all students in infected classed are tested

We also present sample simulation results for a special testing strategy that forces all students in infected classes to be tested after a positive test is found in them. This strategy will allow students to remain in the class if tested negative. Figure 12 and Fig. 13 present the results of these experiments for a sample of tests per day in each class. More daily tests under this strategy allows for significant reduction of infected cases by testing and isolating infected students (Fig. 12).

Figure 13 shows number of tests, number of infected students, and number of students isolated through testing for testing all students in infected classes strategy on day 30 and day 60 of the simulation.

### Validating the simulation results

#### Theoretical validity

In this section we present an estimate on the frequency of testing needed to control outbreaks of COVID-19 in Ontario’s elementary/secondary schools (ages 5-15 yrs). The testing frequency and number of tests refer to a school’s population made up of students & teachers and staff, together. Most importantly, we show that a testing frequency of 3 students per class, per day (as also shown in sections above) is sufficient to keep *R*_*eff*_ ≤ 1 in the school environment, thus preventing outbreaks. In Humphrey et al. [17], a calibrated SEIRL (where L stands for isolated) compartmental model was used to trace the pandemic in various countries. The model gave necessary levels of frequencies for isolating infectious and exposed individuals (via testing and contact tracing) needed to control subsequent waves of the pandemic.

We use here a similar approach but for a school population as follows: We assume a generic elementary/secondary school in Ontario with a population of students (from junior kindergarten to 8-th grade) denoted by *N*_1_ and a population of teachers and staff denoted by *N*_2_. We further denote by subindex-1 the population of students and by subindex-2 the population of teachers & staff. In Prem et al. [13], the authors project average numbers of contacts for 152 countries around the world from the POLYMOD study of Mossong et al. [18] They refer to social contacts that are meaningful in the transmission of influenza and other similar pathogens. The authors give estimates of the average daily number of contacts of children between ages of 5–15 years old in Canada with their peers, during school, to be *c*_11_ =3.46, while the average contact rate of students with their teachers (which we estimate as adults between the ages of 20–65 who work in elementary and secondary school in Ontario) is estimated to be *c*_12_ =0.39 per day. Using a weighted average mix of 5 yrs. population group sizes in Ontario, we compute the average contact rates of teachers with students to be *c*_21_ =0.34 and finally the teachers with other teachers rate to be *c*_22_ =0.31. Since all school contacts take place during school-time (duration 8 h), we obtain the within-school-time contact rate by multiplying the daily number of school contacts by a factor 24 *hrs*/8 *hrs* = 3.

To model the transmission in the two subpopulations of the school, during school days, and disregarding for the time being the coupling of students and teachers/staff populations with the community, we consider the following two sets of differential equations (*j* ∈ {1, 2 }):

$$ \frac{d{s}_j}{dt}=-\left({\beta}_{j1}\ {i}_1+{\beta}_{j2}{i}_2\right){s}_j $$

$$ \frac{d{e}_j}{dt}=\left({\beta}_{j1}\ {i}_1+{\beta}_{j2}{i}_2\right){s}_j-\sigma\ {e}_j-{\kappa_1}^j{e}_j $$

$$ \frac{d{i}_j}{dt}=\sigma\ {e}_j-\left(\gamma +{\kappa_1}^j\right){i}_j $$

$$ \frac{d{\mathrm{l}}_{\mathrm{j}}}{dt}={\kappa}^j\ {i}_j+{\kappa_1}^j{e}_j $$

where *β*_*ji*_ = *c*_*ji*_*prob*_*trns*_, *forall i* = {1, 2} so that *c*_*j*1_ is the average contact rate of a person in group *j* with a child, *c*_*j*2_ is the average contact rate of a person in group *j* with a teacher/staff and where *prob*_*trns*_ is the probability of transmission per contact, taken to be, as in previous sections, 0.14 in Ontario. Finally, *κ*^*j*^ and *κ*_1_^*j*^ are isolation rates due to testing and tracing for the subpopulation *j*. It is understood that the two systems of differential equations describing the subpopulations are to be analyzed together if one wants to extract information on the effective reproductive number *Reff* of the school, as a function of the model parameters.

**Fig. 14:**
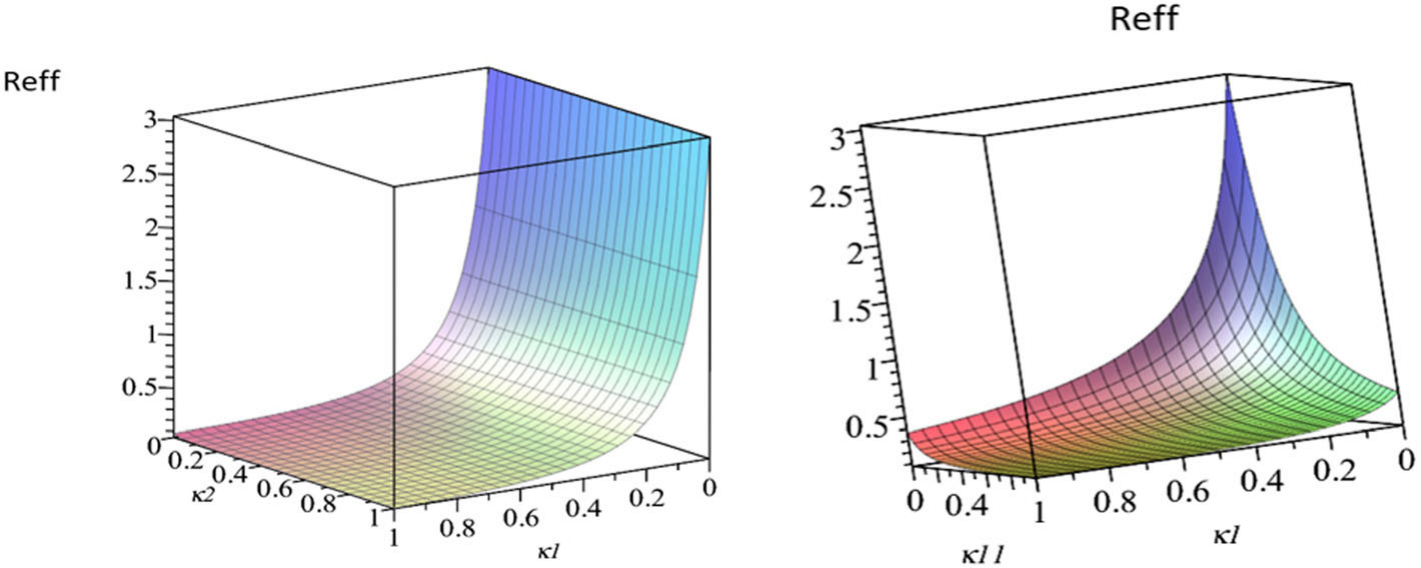
Left Panel: Testing the teachers & staff population matters less in the evolution of the effective reproductive number in the school. Right Panel: Student population drives the disease spread primarily

We assume that the school’s disease free equilibrium (DFE) on September 15, 2020 is given by the value $DFE:= \left(\bar {s_1}:= {N}_1/{N}_1+{N}_2\right),0,0,0,\bar {s_2}:= {N}_2/\left({N}_1+{N}_2\right),0,0,0\Big).$ An analysis around this DFE describing the two subpopulations [19] can be conducted using the next-generation matrix method. With values of contact rates *c*_11_ = 10.38, *c*_12_ = 1.17 , *c*_21_ = 1.029, *c*_22_ = 0.937, $\sigma =1/2.5=0.4;\gamma =0.4,\bar{s}2:= 1-\bar {s_1}, and\ \bar {s_1}=80\%$ we get that: *R*_0_ = *R*_*eff*_(*t* = 0) = 3.03 in the beginning of the pandemic, in the absence of any mitigation measures. Further, we note that *R*_*eff*_ depends primarily on the frequency that the population of students gets tested (*κ*^1^ in the left panel of Fig. 14) but only weakly on the frequency of testing the population of teachers (*κ*^2^ in left panel of Fig. 14).

Therefore we concentrate on getting estimates for the frequency of isolation due to testing (*κ*^1^) and tracing (*κ*^1^_1_) in the population of students, in order to keep the effective reproduction number at 1 (*R*_*eff*_ =1). To be more conservative, we assume that for every isolated student due to testing positive, one exposed student among its contacts can be isolated on average. Therefore, we consider *κ*^1^ = *κ*^1^_1_.

Adopting our assumptions of the paper so far, consider a school with 500 students who represent a proportion of 80% of the school, where the teachers and staff population represents 20%. Then we compute that a frequency of testing of *κ*^1^ = 0.999 =  > 1/*κ*^1^ = 10 *days* will result in the desired *R*_*eff*_ = 1. This means that testing every student every 10 days will ensure an effective reproductive number below the threshold value for school outbreaks. Note that keeping *R*_*eff*_ = 1 is desired when the community transmission and the number of new infections is very low. Otherwise we need to aim for *R*_*eff*_ < 1.

### The effects of facemask and class cohort

Here we show how the theoretical result validates our detailed simulations. In our school of 500 students divided into average class sizes of 25 students, we can, at a minimum, think of testing one student in each class every day. But this is likely not enough to prevent outbreaks, as it amounts to testing every student every 25-th day, which is far below our calculated threshold of testing each student on average every 10 days. It turns out that testing 3 students out of every class daily would amount to testing every child every 8.6 days, and leading to *κ*^1^ = 0.1163 to ensure *R*_*eff*_ < 1. Specifically, we have *R*_*eff*_ = 0.874. The number of tests needed per day in the school in the last scenario would be

$$ 20\ x\ 3=60\  tests\  per\  day=>1800\  tests\  per\  month $$

If we consider that pre-symptomatic students may become symptomatic over the weekend and thus self-isolate come Monday morning, then the student isolation rate *κ*_1_^1^ can be improved by at least a 2/7 ratio per week, that is to say, a 2/49 ratio per day: *κ*_1_^1^ = *κ*^1^ + 2/49 which would imply that the frequency of testing to maintain *R*_*eff*_ = 1 would be *κ*^1^ = 12.45 *days*. This would mean a decrease to a frequency of 2 tests per class, per day, for a total of

$$ 20\ x\ 2=40\  tests\  per\  day=>1200\  tests\  per\  month. $$

Next, we look at how we can account for mask wearing (and other social-distancing) policies voted into effect by many school boards in Ontario, as well as the policy of class cohorts, i.e. the reduced contacts that individual classes can have with other classes in the school. In general, both of the measures above will affect the effective contact rates, *β*_*ij*_, albeit from two differing angles: mask wearing reduces the per-contact probability of transmission *prob*_*trns*_, while class cohorts restrictions reduce the average contacts *c*_*ij*_, *i*, *j* ∈ {1, 2}. We can incorporate both effects in our estimate by considering the effective contact rates as:

$$ {\beta}_{ij}=\left(1- cohor{t}_{red}\right)\ {c}_{ij}\ \left(1- mas{k}_{red}\right)\  pro{b}_{trns} $$

where *cohort*_*red*_ and *mask*_*red*_ are the notations for the reduction factors described above. The mask wearing reduction factor is taken to be in a range of [0.3, 0.8] [20] where 0.3 effectiveness is the level of a paper mask or 1-layer mask, while 0.8 and higher are surgical masks and N95 masks, which are not typically available to everyday students). Specifically, we take it here equal to 0.3. Cohort reduction is taken to be 0.05 for exemplification purposes. Both values are fairly conservative. Under the new assumptions, we compute that the frequency of isolating students due to testing and tracing is to be 17.7 *days* (*κ*^1^ = 0.0565) for an effective reproductive number of *R*_*eff*_ = 1. This would amount to roughly 1.5 students needed to be tested per class per day (1 student in half the classes, 2 students in the other half per day, then switch the next day). This would mean a decrease to a frequency of 1.5 tests per class, per day, for a total of

$$ 20\ x\ 1.5=30\  tests\  per\  day=>900\  tests\  per\  month. $$

We note that the range of values for the *cohort*_*red*_ reduction factor is not yet known. An increase from 0.05 to 0.1 would amount to a further decrease to testing every student every 19.5 days, etc. An increase in *mask*_*red*_ to 0.4 (from 0.3) amounts to testing every student every 26 days, which is the equivalent to testing 1 student in every class, per day, for a total of

20 *tests per day* =  > 600 *tests a month* for an *R*_*eff*_ = 1.

All these scenarios are consistent with the results of our simulations in Section 4. In fact, they represent an upper limit on the number of daily tests needed. Our agent-based simulations show a clearer picture of the proposed testing process and take into consideration a much more detailed transmission dynamics.

## Discussion

In this simulation we have not included testing accuracy issues that are also very important [10]. Rapid tests that expedite the testing process, if used, may have larger accuracy issues that need to be considered. The relevant parameter can be added to this simulation tool to account for test accuracy level. Similarly, this simulation in its current form does not factor in contact tracing so that if a student test becomes positive, all students in contact with the infected student are self-isolated which probably means the closure of the whole class. This again can be added to this simulation tool. One possibility would be to design the simulation so that if a student’s test in a class becomes positive all students in that class are tested.

If rapid tests are available, the idea of pool testing can also be incorporated in this model. Pool testing adopts this tree-search algorithm to testing specimen aiming to increase the number of people tested using fewer number of tests. In pool testing, several specimens collected from different people are mixed and one test is done to find if that mix of specimens indicates an infection. In that case, the sources of that mix are divided into two groups, mixing the specimens in two or more different groups, to perform the test on the new – drilled-down mixes – to find which subset(s) of the specimen mixes shows an infection. This drill-down process is continued until the infected specimen(s) is (are) identified.

Finally, although this simulation focuses on schools, it can be adapted for similar situations where a number of people stay close to each other in one complex such as universities and workplaces.

## Conclusion

In this paper, we presented an agent-based simulation tool for COVID-19 testing at schools. The model allows users to set several parameters and investigate the impacts of different testing approaches for controlling the outbreak in schools. The simulation is flexible in allowing different school sizes (based on number of classes and students in each class) and different parameter settings according to the local disease situation and testing policies.

This simulation is not a means for advocating school testing, rather it aims to help making such decisions. By using this tool, public health decisionmakers and school districts officials can decide whether and to what extent school testing would help them to control the outbreak, while being able to calculate the per capita costs of conducting tests at schools.

## Data Availability

The data used in this study are generated by the simulation models developed in this study. The simulation tool is available at: https://cloud.anylogic.com/model/a7c4411e-064e-4283-a93c-b0b27e0430ee

## Abbreviations

SARS-CoV-2: Severe Acute Respiratory Syndrome Coronavirus 2
COVID-19: Corona Virus Disease 2019
SEIR: Susceptible, Exposed, Infectious, Recovered
ECDC: European Centre for Disease Prevention and Control (ECDC),

## References

[1] European Centre for Disease Prevention and Control (ECDC) (2020). Objectives for COVID-19 testing in school settings.

[2] Panovska-Griffiths J, Kerr CC, Stuart RM, et al. Determining the optimal strategy for reopening schools, the impact of test and trace interventions, and the risk of occurrence of a second COVID-19 epidemic wave in the UK: a modelling study. Lancet Child Adolesc Health. 2020; published online Aug. 10.1016/S2352-4642(20)30250-9. 10.1016/S2352-4642(20)30250-9 PMC7398659 32758453

[3] World Health Organization. Considerations for school-related public health measures in the context of COVID-19: World Health Organization; 2020. https://www.who.int/publications/i/item/considerations-for-school-related-public-health-measures-in-the-context-of-covid-19 (Accessed 10 Sept 2020).

[4] Centers for Disease Control and Prevention (CDC) (2020). Screening K-12 Students for Symptoms of COVID-19: Limitations and Considerations.

[5] Centers for Disease Control and Prevention (CDC) (2020). Cleaning, Disinfection, and Hand Hygiene in Schools – a Toolkit for School Administrators.

[6] Government of Canada (2020). Risk mitigation tool for child and youth settings operating during the COVID-19 pandemic.

[7] Government of Ontario (2020). Archived - approach to reopening schools for the 2020–2021 school year.

[8] Service R. Radical shift in COVID-19 testing needed to reopen schools and businesses, researchers say. Science. 2020; published online Aug 3. 10.1126/science.abe1546.

[9] Lazer D, Santillana M, Perlis RH, et al. The State of the Nation: A 50-State COVID-19 Survey Report #8: Failing the Test. Open Sci Framework. 2020. 10.31219/osf.io/gj9x8.

[10] Gressman PT, Peck JR (2020). Simulating COVID-19 in a university environment. Math Biosci.

[11] Frank K, Arim R (2020). Canadians’ willingness to get a COVID-19 vaccine when one becomes available: what role does trust play?.

[12] Kirkey S. Should random COVID-19 tests for kids and teachers be part of our back-to-school plans? Natl Post. 2020; published online Aug 20. https://nationalpost.com/news/should-random-covid-19-tests-for-kids-and-teachers-be-part-of-our-back-to-school-plans (Accessed 10 Sept 2020).

[13] Prem K, Cook AR, Jit M (2017). Projecting social contact matrices in 152 countries using contact surveys and demographic data. PLoS Comput Biol.

[14] Abdollahi E, Haworth-Brockman M, Keynan Y, Langley JM, Moghadas SM (2020). Simulating the effect of school closure during COVID-19 outbreaks in Ontario, Canada. BMC Med.

[15] Tang B, Scarabel F, Bragazzi NL (2020). De-escalation by reversing the escalation with a stronger synergistic package of contact tracing, quarantine, isolation and personal protection: feasibility of preventing a COVID-19 rebound in Ontario, Canada, as a case study. Biology.

[16] Byrne AW, McEvoy D, Collins A, et al. Inferred duration of infectious period of SARS-CoV-2: rapid scoping review and analysis of available evidence for asymptomatic and symptomatic COVID-19 cases. Epidemiology. 2020. 10.1101/2020.04.25.20079889. 10.1136/bmjopen-2020-039856 PMC7409948 32759252

[17] Humphrey L, Thommes EW, Fields R, Hakim N, Chit A, Cojocaru MG. A path out of COVID-19 quarantine: an analysis of policy scenarios. medRxiv. 2020. 10.1101/2020.04.23.20077503.

[18] Mossong J, Hens N, Jit M (2008). Social contacts and mixing patterns relevant to the spread of infectious diseases. PLoS Med.

[19] van den Driessche P (2017). Reproduction numbers of infectious disease models. Infect Dis Modelling.

[20] Wilson AM, Abney SE, King M-F (2020). COVID-19 and use of non-traditional masks: how do various materials compare in reducing the risk of infection for mask wearers?. J Hosp Infect.

